# Addressing regional relationships between white‐tailed deer densities and land classes

**DOI:** 10.1002/ece3.8084

**Published:** 2021-08-31

**Authors:** Brice B. Hanberry

**Affiliations:** ^1^ USDA Forest Service Rocky Mountain Research Station Rapid City South Dakota USA

**Keywords:** benefits, broadleaf forest, crops, edge, extreme gradient boosting, fire, land use

## Abstract

White‐tailed deer (*Odocoileus virginianus*) populations have recovered to about 30 million animals in the United States, but land cover has changed during the interval of recovery. To address the relationship between deer densities and current land cover at regional scales, I applied random forests and extreme gradient boosting classifiers to model low and high deer density classes, at two different thresholds (5.8 and 11.6 deer/km^2^), and land classes in three regions during approximately 2003. For low and high deer density classes divided at 5.8 deer/km^2^, deciduous broadleaf forest overall was the most influential and positive variable in the central east and central regions and crop and pasture were the most influential and negative variables in the southeast region. Deer density increased with area of deciduous and mixed forests, woody wetlands, and shrub in all regions. Deer density decreased with area of crop, developed open space, and developed low and medium residential density in all regions. For density classes divided at 11.6 deer/km^2^, deer density had the strongest relationship with woody wetlands in the central east region, mixed and deciduous forest in the southeast region, and woody wetlands and herbaceous vegetation in the central region. Deer density increased with deciduous and mixed forests, woody wetlands, and shrub in all regions. Conversely, deer density decreased with herbaceous vegetation, crop, and developed low residential densities in all regions. Therefore, at regional scales, deer overall occurred at greater densities in forests and woody wetlands and lower densities in agricultural and residential development, which did not appear to support more deer. Deer preference for forests does result in damage to forest products, but alternatively, some may consider that deer provide important socioeconomic and ecological services by reducing number of small trees, particularly in the absence of other disturbances that historically controlled tree biomass.

## Graphical abstract

At regional scales, deer occurred at greater densities in forests and lower densities in agricultural and residential development, which did not improve habitat by supporting more deer. Deer preference for forests does result in damage to forest products. However, deer provide important socioeconomic and ecological services by reducing number of small trees, particularly in the absence of other disturbances that historically controlled tree biomass.

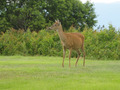

## 1 INTRODUCTION

**FIGURE 1:**
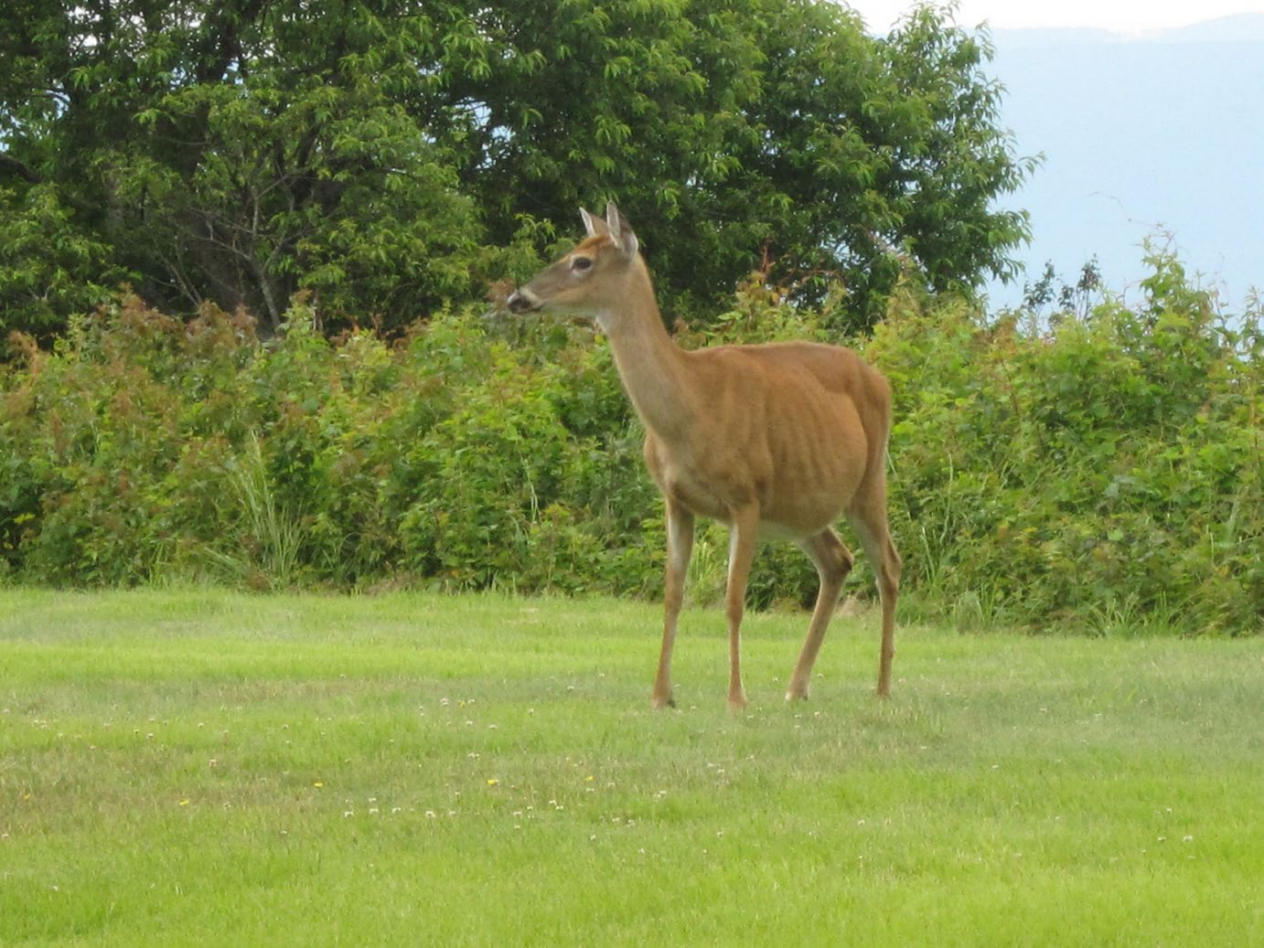
White‐tailed deer (*Odocoileus virginianus*) in West Virginia. Photo courtesy of B. Hanberry

White‐tailed deer (*Odocoileus virginianus*; Figure 1) populations have recovered steadily throughout most of the United States to about 30 million animals after population reductions to 300,000–500,000 between the years 1850 and 1900 (Hanberry & Hanberry, 2020a, 2020b; McCabe & McCabe, 1984; VerCauteren, 2003). However, during this interval, historical forests and resources have changed. Overstory disturbance was rare in the past (Lorimer, 2001; Lorimer & White, 2003; Seymour et al., 2002) and mature forests of savannas and woodlands covered most of the eastern United States (i.e., predominantly longleaf pine [*Pinus palustris*] in the southern half of the southeastern United States, shortleaf pine [*Pinus echinata*] and upland oaks [e.g., fire‐tolerant species such as *Quercus alba*] in the northern half of the southeastern United States, and mostly upland oaks in the central eastern United States; Hanberry et al., 2018). By definition, savannas and woodlands contain a bilayer of overstory trees and understory grasslands, with abundant and diverse forbs, which means that trees and herbaceous vegetation spatially coexisted (Hanberry et al., 2020). Less young tree browse is available in open forests due to small tree removal by frequent surface fires that maintained herbaceous growing space, but burned areas produce herbaceous vegetation regrowth with greater protein content that is preferred for foraging (“pyric herbivory”; Fuhlendorf et al., 2009). Additionally, new forage is available earlier in the growing season after dead vegetative ground cover is removed by fire.

Compared to historical forests, forage overall has not likely increased in forests and clearings that are separated into ephemeral patches. Due to fragmentation from different land uses and frequent overstory disturbance by cutting for harvest, current landscapes have sharp edges at the juxtaposition between transient clearings with herbaceous plants and closed successional forests, which have limited herbaceous resources and instead have great tree densities (Hanberry et al., 2018; Pan et al., 2011). Nonetheless, white‐tailed deer have become successful in current landscapes of fragmented forests and pasture, agricultural fields, and residential development. Agriculture is believed to have improved deer habitat (Côté et al., 2004), and indeed, deer cause more damage to crops than any other wildlife species (Conover, 1997).

Researchers have started to analyze habitat use by deer at scales larger than stands, specifically focusing on configuration of contrasting land classes that provide different resources in space and time (Walter et al., 2009). Deer densities and land classes from satellite imagery have been modeled at landscapes as large as one U.S. state (i.e., Illinois; Roseberry & Woolf, 1998) and deer densities and land inventories have been modeled for the southern United States (Flather et al., 1989), but otherwise models appear to remain rare at most scales. Regional modeling can help support anecdotal knowledge and stand scale studies, such as the apparent benefit of agriculture and residential development to deer, or conversely, highlight discrepancies that may need further study. This additional line of evidence can inform large‐scale deer management.

**FIGURE 2:**
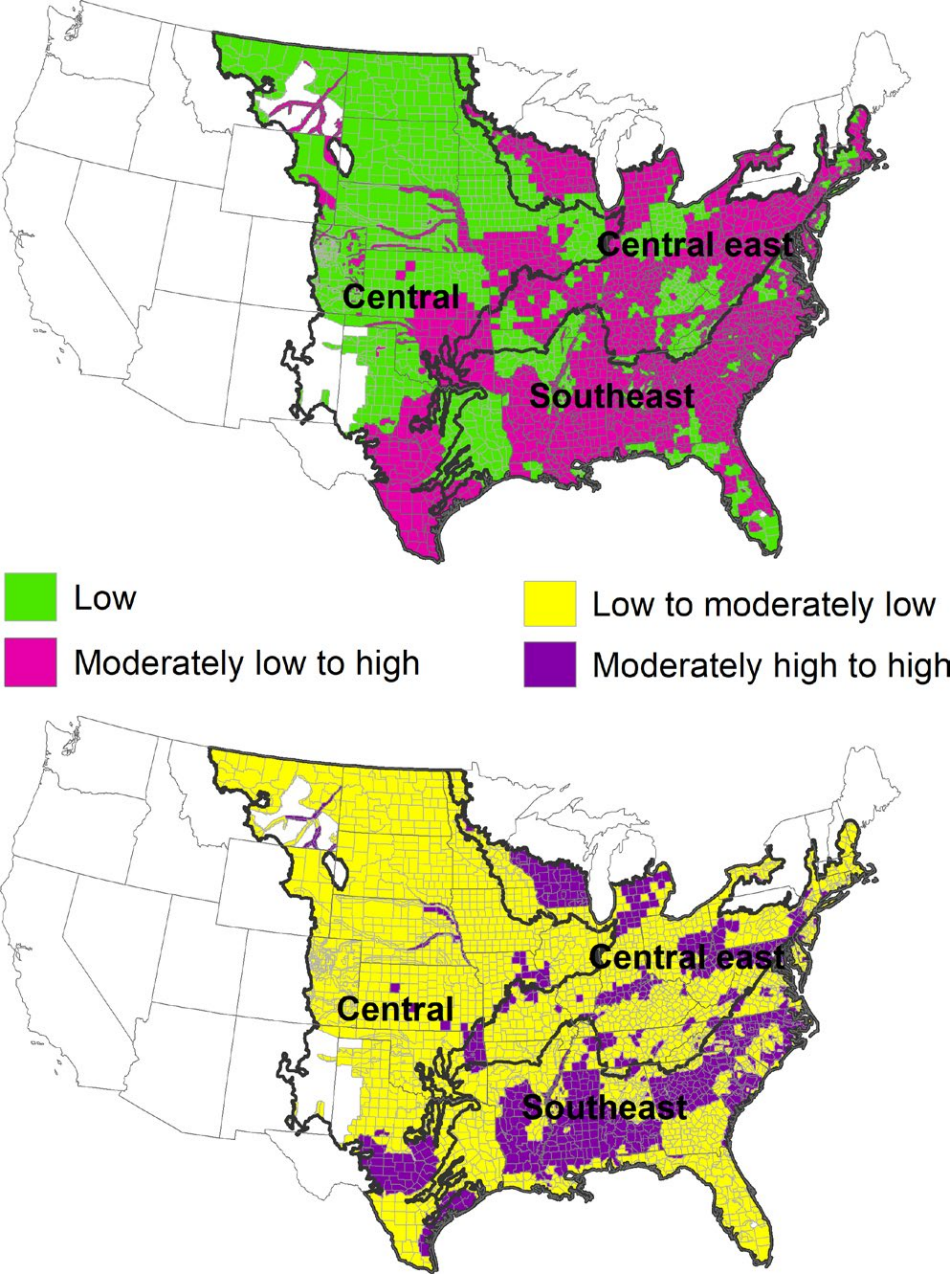
Regions and white‐tailed deer densities: low‐density class (<5.8 deer/km^2^), moderately low to high‐density class (≥5.8 deer/km^2^), low and moderately low‐density class (<11.6 deer/km^2^), and moderately high and high‐density class (≥11.6 deer/km^2^)

Research has been limited by the availability of spatial deer data. However, digitized layers from archival data comprised of four deer density classes assigned by wildlife agencies are now available for the conterminous United States (Hanberry & Hanberry, 2020a). Deer population sizes remain difficult to estimate and have great uncertainty, even with current modeling methods, so that some states have discontinued population estimates (Adams & Ross, 2015). Nonetheless, estimates from harvest data and deer surveys by state wildlife agencies represent the only consistent source of deer densities for management and research. All assignments into each of four deer density classes may not be correct, but it is likely that most assignments were accurate, particularly given the need to only differentiate between basic density classes (Figure 2), rather than more complex estimates. Additionally, densities cumulatively are consistent with state‐wide population reports (Hanberry & Hanberry, 2020a). Similarly to the possibility that some of the deer density classes may be incorrect, species distribution models based on presence and absence samples rely on “pseudoabsences,” because it is uncertain if absences are true absences. Despite uncertainty, these models tend to be robust and informative.

To expand current information about the relationship between land classes and white‐tailed deer, my objective was to model the relationship between deer density classes and land classes in the United States during approximately 2001–2005. White‐tailed deer are present continuously throughout most of the eastern and central regions of the United States (Figure 2) and accordingly, deer‐vegetation models need to be based on different densities rather than presence and absence, at least at landscape scales. According to a comprehensive analysis of 433 studies in temperate forests, densities of 5.3 white‐tailed deer per km^2^ reduce tree regeneration, 6.5 white‐tailed deer per km^2^ affect forest structure, and 11.6 white‐tailed deer per km^2^ impair forest functioning (Ramirez et al., 2018). These thresholds accorded well with the white‐tailed deer density classes assigned by wildlife agencies. I modeled low and high deer density classes divided by the threshold of 5.8 deer/km^2^, comparing low densities with minimal potential ecological impacts to moderately low to high densities, and also modeled low and high deer density classes divided by 11.6 deer/km^2^, comparing low to moderately low densities to moderately high to high densities with greatest impact. This study will verify if deer indeed occur at greater densities in forests, and if so, which types of forest, and help establish the influence of agricultural and residential development on deer densities.

## 2 METHODS

I separated the conterminous United States into eastern forests and central grasslands. I subdivided the eastern region into the central east and southeast ecoregions (Figure 2; hereafter each of the three extents is considered a region). The regions generally followed ecological divisions, but I moved the forested Oklahoma Cross Timbers and Texas savannas from the central grasslands to eastern forests (Cleland et al., 2007).

I used 2001–2005 deer density classes for modeling and to calculate deer number per region (Figure 2; Hanberry & Hanberry, 2020a, available at https://www.fs.usda.gov/rds/archive/catalog/RDS‐2019‐0053). Deer densities were estimated by wildlife agencies in four density classes: <5.8, 5.8–11.6, 11.6–17.4, and >17.4 deer/km^2^. Spatial units were by county or divisions of the county.

I grouped two classes of low and high deer densities for modeling at two different thresholds. Modeling only two deer density classes also reduced error in density class assignments due to only needing to differentiate between two classes. I retained the low‐density class (<5.8 deer/km^2^) and collapsed the other three density groups into one moderately low to high‐density class (≥5.8 deer/km^2^). Additionally, I grouped the low and moderately low‐density classes (<11.6 deer/km^2^) to compare with the combined moderately high and high‐density classes (≥11.6 deer/km^2^). Sample size for the greatest density class was too small (≤100) to model well and even combined with the moderately high‐density class, the sample size remained low at 240 samples for the central region.

The National Land Cover Database for the United States recently was revised to update all previously released versions of land cover, which included year 2004 (Homer et al., 2020). For this model, variables were twelve land cover variables (developed open space, developed low intensity, developed medium intensity, deciduous broadleaf forest, evergreen needleleaf forest, mixed forest, shrub, herbaceous, crop, pasture, woody wetlands, herbaceous wetlands) and two combinations of land cover: vegetation conducive to fire (evergreen forest, herbaceous, and herbaceous wetlands) and vegetation not conducive to fire (crop, pasture, and developed open space, Hanberry, 2020a; Table 1). I used these combinations because fire is a disturbance type similar to deer browsing. I calculated mean values of land class percent area for the two deer densities by region.

**TABLE 1:** Percent area of land class variables (Fire = Evergreen forest + Herbaceous + Herbaceous wetlands, Fire minus = Pasture + Crop + Developed open) by region and deer density class (low‐density class <5.8 deer/km^2^, moderately low to high‐density class ≥5.8 deer/km^2^, low and moderately low‐density class <11.6 deer/km^2^, and moderately high and high‐density class ≥11.6 deer/km^2^)

	Central east low	Central east moderately low to high	Southeast low	Southeast moderately low to high	Central low	Central moderately low to high
Developed open	5.86	5.64	5.68	5.04	3.02	2.84
Developed low	3.59	3.08	3.72	2.45	1.58	1.48
Developed medium	1.97	1.34	1.40	0.84	0.57	0.46
Deciduous forest	29.75	36.48	10.22	12.86	3.21	7.88
Evergreen forest	1.81	1.65	11.22	16.07	0.64	2.28
Mixed forest	7.18	7.44	6.41	7.55	0.73	0.82
Shrub	0.48	0.54	2.36	4.07	8.97	14.12
Herbaceous	1.58	1.80	3.62	3.83	25.61	21.48
Pasture	13.38	15.85	12.98	10.12	8.39	12.59
Crop	25.25	17.15	17.00	12.28	41.89	29.39
Woody wetland	2.60	3.27	14.48	16.52	1.28	2.08
Herbaceous wetland	1.08	0.89	4.06	2.34	1.63	1.56
Fire^a^	4.46	4.33	18.90	22.24	27.89	25.33
Fire minus^b^	44.48	38.65	35.67	27.43	53.30	44.82
	**Central east low and moderately low**	**Central east moderately high to high**	**Southeast low and moderately low**	**Southeast moderately high to high**	**Central low and moderately low**	**Central moderately high to high**
Developed open	5.61	5.90	5.59	4.70	2.95	2.85
Developed low	3.32	3.04	3.21	2.19	1.58	1.32
Developed medium	1.67	1.24	1.14	0.77	0.51	0.54
Deciduous forest	32.87	37.72	11.67	12.88	5.08	6.87
Evergreen forest	1.79	1.50	12.95	17.30	0.90	3.90
Mixed forest	7.34	7.41	6.50	8.25	0.76	0.82
Shrub	0.51	0.54	3.28	4.10	9.07	22.62
Herbaceous	2.13	0.97	4.03	3.44	25.55	14.57
Pasture	14.57	16.22	11.17	10.40	10.03	11.92
Crop	21.25	16.16	15.53	10.81	38.23	25.52
Woody wetland	2.64	3.92	15.45	16.72	1.45	2.67
Herbaceous wetland	0.92	1.00	3.26	2.14	1.45	2.37
Fire^a^	4.83	3.47	20.25	22.88	27.89	20.84
Fire minus^b^	41.42	38.28	32.29	25.91	51.20	40.29

Note

Light gray shading shows land classes that increased with deer density class.

^a^ Fire = Evergreen forest + Herbaceous + Herbaceous wetlands.

^b^ Fire minus = Pasture + Crop + Developed open.

For this modeling, I applied random forests and extreme gradient boosting classifiers. Both random forests and extreme gradient boosting are ensemble methods that build a relationship from numerous classifications. The random forests classifier employs bagging to subsample the data and then creates independent classifications that are averaged. Extreme gradient boosting sequentially develops classifications and each subsequent classification is trained to improve the already trained ensemble.

I employed the caret R package, which trained the model with 10‐fold cross‐validation (Kuhn, 2008; R Core Team, 2020). Validation occurred on separate testing data (25% for this modeling), with withheld known classes, to determine how well the classifier assigned classes using explanatory variables. I set prevalence, or the sample number of the density classes, to equal. Samples were 639 of each class (i.e., 1,278 total) in the central east, 490 in the southeast, and 765 in the central region for the low‐density class (<5.8 deer/km^2^) and moderately low to high‐density class (≥5.8 deer/km^2^). Samples were 739 of each class (i.e., 1,278 total) in the central east, 851 in the southeast, and 242 in the central region for the low and moderately low‐density class (<11.6 deer/km^2^) and moderately high and high‐density class (≥11.6 deer/km^2^).

Lastly, I modeled the relationship between low and high deer density classes at the 5.8 deer/km^2^ threshold and land classes in the three regions, repeated by modeling the relationship between low and high deer density classes at the 11.6 deer/km^2^ threshold and land classes in the three regions. I reported overall accuracy, or the number of predicted classes that matched recorded class assignments divided by the total number of samples. I examined variable importance (if the influence relative value was >50, out of a 100 scale) and compared the most important variables using mean values of land class percent area for the two deer densities by region.

## 3 RESULTS

In total, these U.S. regions with deer covered approximately 4.4 million km^2^ and contained about 24,860,000 deer during approximately 2003 (i.e., deer estimates ranged from years 2001 to 2005). The central region, which covered approximately 2 million km^2^ where deer were present, contained about 7,220,000 deer. The central east region, approximately 1.2 million km^2^, contained about 7,760,000 deer. The southeast region, approximately 1.2 million km^2^, contained about 9,880,000 deer.

The classifiers generally had trouble distinguishing areas of low and high deer density, with accuracy ranging from 0.60 to 0.75 (Table 2). In contrast to presence–absence models, deer are present throughout the modeling extent and the two classes are continuous. Areas of low deer density have the potential to be high deer density.

**TABLE 2:** Accuracy of the low and high deer density classes (low‐density class <5.8 deer/km^2^, moderately low to high‐density class ≥5.8 deer/km^2^, low and moderately low‐density class <11.6 deer/km^2^, and moderately high and high‐density class ≥11.6 deer/km^2^) for random forests and extreme gradient boosting classifiers based on test set (25% of observations) for modeled training data in the central eastern and southeastern ecoregions of the eastern United States and the central United States

	Central east	Southeast	Central
Random forests			
Accuracy of classes divided at 5.8 deer/km^2^	0.67	0.64	0.75
Accuracy of classes divided at 11.6 deer/km^2^	0.67	0.69	0.71
Extreme gradient boosting			
Accuracy of classes divided at 5.8 deer/km^2^	0.65	0.65	0.72
Accuracy of classes divided at 11.6 deer/km^2^	0.60	0.70	0.71

Nonetheless, the models indicated importance for land classes where deer occurred at two different densities (Table 3). Influential land classes remained relatively consistent between the two classifiers. Influential land classes varied to some extent with region and the increased division in deer density classes from 5.8 deer/km^2^ (i.e., low‐density class and moderately low to high‐density class) to 11.6 deer/km^2^ (i.e., low and moderately low‐density class and moderately high and high‐density class). In particular, more variables were influential (value >50 out of 100) in models differentiating the low and moderately low‐density class from moderately high and high‐density class, perhaps indicating that these density classes were similar and difficult to separate without numerous variables.

**TABLE 3:** Most important variables and importance value (if ≥50) for random forests and extreme gradient boosting (egb) models of deer densities (low‐density class <5.8 deer/km^2^, moderately low to high‐density class ≥5.8 deer/km^2^, low and moderately low‐density class <11.6 deer/km^2^, and moderately high and high‐density class ≥11.6 deer/km^2^) by region

Central east low and moderately low to high				Central east low and moderately low, moderately high and high			
Random forests		egb		Random forests		egb	
Fire minus^a^	100	Deciduous forest	100	Woody wetland	100	Woody wetland	100
Deciduous forest	89	Developed open	54	Fire minus	62	Fire^b^	86
Developed open	59	Pasture	52	Herbaceous	54	Mixed forest	77
Mixed forest	52	Developed medium intensity	51			Herbaceous	72
Developed low intensity	50					Shrub	57
						Developed open	56
						Deciduous forest	51
						Herbaceous wetland	51
						Pasture	50
**Southeast low and moderately low to high**				**Southeast low and moderately low, moderately high and high**			
**Random forests**		**egb**		**Random forests**		**egb**	
Pasture	100	Crop	100	Mixed forest	100	Deciduous forest	100
Crop	83	Pasture	57	Deciduous forest	94	Mixed forest	71
Shrub	62	Developed open	57	Woody wetland	85	Pasture	68
Fire minus	62	Herbaceous	54	Herbaceous	73	Herbaceous	64
				Pasture	69		
**Central low and moderately low to high**				**Central low and moderately low, moderately high and high**			
**Random forests**		**egb**		**Random forests**		**egb**	
Deciduous forest	100	Deciduous forest	100	Herbaceous	100	Woody wetland	100
		Shrub	59	Shrub	86	Herbaceous	89
				Woody wetland	76	Herbaceous wetland	81
				Crop	75	Developed open	78
				Herbaceous wetland	70	Evergreen forest	71
				Developed open	69	Crop	65
				Evergreen forest	67		

Note

Fire = Evergreen forest + Herbaceous + Herbaceous wetland, Fire minus = Pasture + Crop + Developed open.

^a^ Fire minus = Pasture + Crop + Developed open.

^b^ Fire = Evergreen forest + Herbaceous + Herbaceous wetlands

### 3.1 Low‐density class compared to moderately low to high‐density class

For influential land classes at the 5.8 deer/km^2^ division in deer density classes, the variables that were most influential (value 100 out of 100) in the central east were deciduous broadleaf forest and the combination of crop, pasture, and developed open space. In the southeast region, crop and pasture were most influential. In the central region, deciduous broadleaf forest was most important.

**FIGURE 3:**
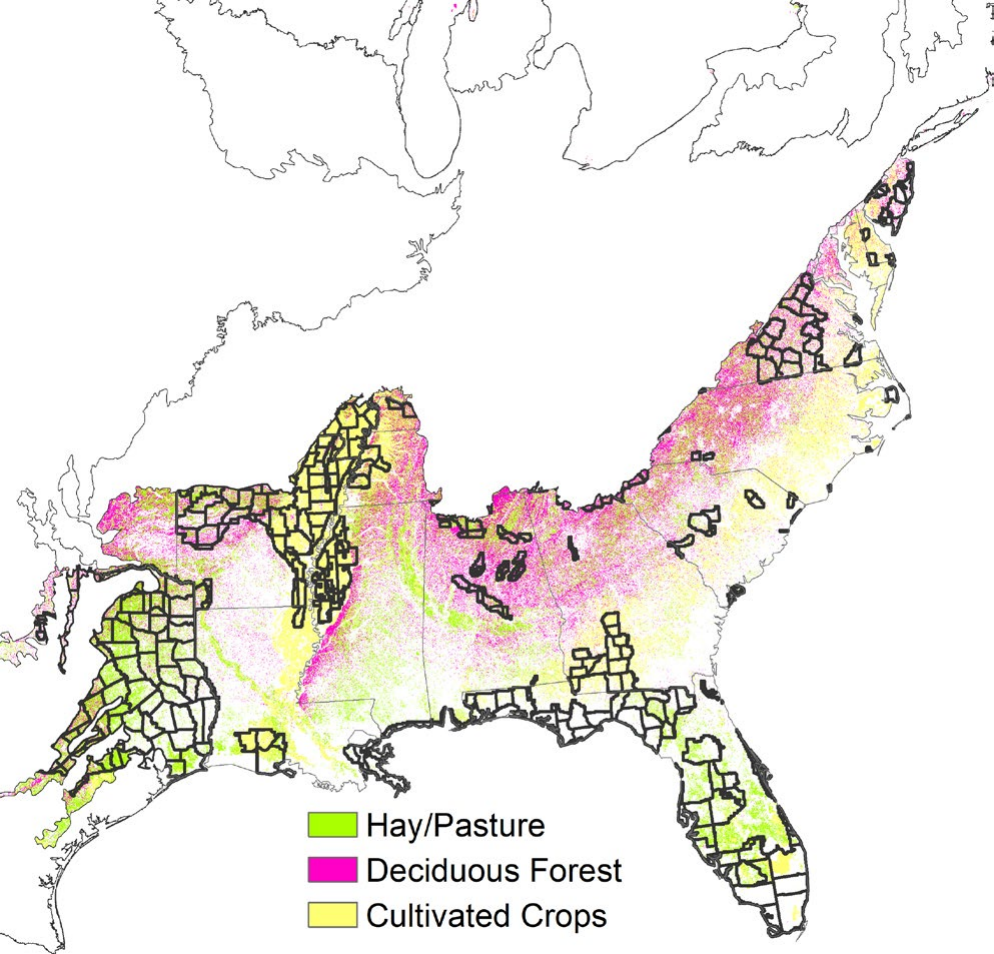
Influential land classes and low deer densities (black outlined counties) in the southeast region

Percent area of deciduous broadleaf forest was greater for the high deer density class in the three regions (Table 1, Figure 3). Percent area of mixed forest was greater for the high‐density class in the three regions but mixed forest was not very influential, with the greatest value of 52 out of 100 for the random forests classifier in the central east. The percent area of evergreen forest was not influential, but greater for the high‐density class in the southeast and central regions. Shrubs and woody wetlands were other positive variables greater for the high‐density class in the three regions, but not very influential. Herbaceous vegetation was not influential, with the greatest value of 54 out of 100 for the extreme gradient boosting classifier in the southeast region, but greater for the high‐density class in the southeast and central east regions.

Crop; the combination of crop, pasture, and developed open space; developed open space; and developed low and medium intensity (residential density) all decreased in percent area in the high‐density class compared to the low‐density class for the three regions. Herbaceous wetlands also decreased in percent area with deer density but was not influential. Pasture percent area increased in the central east and central regions but decreased where it was most influential in the southeast region.

### 3.2 Low and moderately low‐density class compared with moderately high to high‐density class

For influential land classes at the 11.6 deer/km^2^ division in deer density classes, the variable that was most influential (value 100 out of 100) in the central east was woody wetlands. In the southeast region, mixed and deciduous broadleaf forest was most important. In the central region, herbaceous vegetation and woody wetlands were most important.

Percent area of deciduous broadleaf and mixed forest was greater for the high deer density class in the three regions, although these land classes were most influential in the southeast region (Tables 1, 3). The percent area of evergreen forest was greater for the high‐density class in the southeast and central regions, but only influential in the central region. Woody wetlands increased for the high deer density class in the three regions and was influential in the central east and central regions. Percent area of shrub was greater for the high‐density class in the three regions and influential in the central region.

Herbaceous vegetation; crop; the combination of crop, pasture, and developed open space; and developed low intensity (residential density) all decreased in percent area in the high‐density class compared to the low‐density class for the three regions. Herbaceous vegetation was influential in all regions, particularly in the central region. Pasture was influential in the southern region and crop was influential in the central region. The combination of crop, pasture, and developed open space and the combination of evergreen forest, herbaceous, and herbaceous wetlands were influential in the central eastern region.

## 4 DISCUSSION

Based on modeled relationships between deer density classes and land cover in three regions of the United States, white‐tailed deer occurred at greater densities in forests and woody wetlands. Deer occurred at lesser densities in nonforests. The lowest densities (<5.8 deer/km^2^) corresponded with crop, pasture, and developed open space, whereas herbaceous vegetation corresponded with lower densities at the next density class level (<11.6 deer/km^2^). Results were consistent with the other landscape models of deer and vegetation relationships. In Illinois, forest cover of closed deciduous forests was the primary model variable to determine deer distributions compared to other forest types and agricultural crops (Roseberry & Woolf, 1998). Refinements such as patch size and distance, harvest rate, and hunter density did not improve the model. Similarly, for the southern United States, cropland had a negative influence on deer density where cropland was dominant although cropland had a positive influence where forest cover was dominant (Flather et al., 1989).

Deciduous broadleaf forest was the most influential and positive variable for deer densities ≥5.8 deer/km^2^ in the central east and central regions, whereas deciduous and mixed deciduous and broadleaf forests were the most influential and positive variable for deer densities ≥11.6 deer/km^2^ in the southeast region. For deer densities ≥11.6 deer/km^2^ in the central east and central regions, woody wetlands became influential; woody wetlands can consist of broadleaf tree species and preferred (nonpine) needleleaf browse and the wetlands may add an extra cover component. Deer use broadleaf trees for cover and browse, with little preference for pine trees as browse, and broadleaf forests are considered better than other forest types for deer (McShea, 2012; Roseberry & Woolf, 1998; Warren & Hurst, 1981). The central east region is comprised primarily of broadleaf forest (Table 1). The central region overall is nonforested, with trees historically limited to riparian networks, steep slopes, or rocky locations, but both broadleaf and conifer trees have encroached into grasslands during the past century (Hanberry, 2020b). Conversely, the southeast region contained more pine forests and greater deer densities than other regions (almost 10 million deer in an area equal or smaller than the other regions, see also Table 1), albeit pine forests are primarily commercial stands managed on short rotations of 20–25 years, which produces landscapes of about 25% young forest (Hanberry & Thompson, 2019).

At regional scales, the influence of agricultural development was negative and strong, particularly in the southeast region. This may be because crop and pasture are less adjacent to forests in the Southeast. For example, the primarily agricultural Lower Mississippi Alluvial Valley of about 11 million ha is within the southeast region and contains low‐density deer areas (Figure 3). The 3.1 million ha of forest fragments generally occur in lower elevation areas that are more flood‐prone and in discrete patches not intermixed with agriculture (Elliott et al., 2020). The other large extent in the region was in Texas, which likely contained a low density of trees historically and has been converted to pasture, with few trees. Flather et al. (1989) noted these regions as being dominated by agricultural land, which negatively affects deer densities. VerCauteren and Hygnstrom (2011) also stated that deer were more abundant where forest is at least 25% of landscapes.

Residential development also was greater at lower deer densities, which only manifested in modeling as a weak influence in the central eastern region. Developed low and medium residential density decreased in percent area for the high‐density deer class compared to the low‐density deer class at the 5.8 deer/km^2^ threshold for the three regions. Developed low and medium residential density decreased in percent area for the high‐density class compared to the low‐density class at the 11.6 deer/km^2^ threshold for the central east and southeast regions. At the 11.6 deer/km^2^ threshold for the central region, developed low residential density decreased and remained at similar percentages for the medium residential density as deer densities increased. Further research to corroborate this negative relationship between residential development and deer densities remains necessary.

Influential land classes remained relatively consistent between the two classifiers, but the classifiers did not have the best accuracy (0.60–0.75) in differentiating two density classes, indicating that low‐density areas have the potential to be high‐density areas and that of course, a variety of other factors influence deer densities. Additionally, due to the difficulty in estimating deer densities, error may have occurred in deer density assignments by state wildlife agencies. However, some of this error was reduced by collapsing four density classes into two classes. This amount of error may be equivalent to pseudoabsence assignments for species distribution modeling.

Deer and surface fire both are disturbances that have removed understory vegetation for millions of years, resulting in interactions between these disturbances and land classes. Based on modeling of current deer densities and land classes, consumption of biomass by deer and fire have some overlap in wildlands, albeit not all land classes were influential in models. Deer densities and fire occurrences increased in deciduous forest in the central east and central regions (Hanberry, 2020a). Deer densities and fire occurrences increased in evergreen forest, herbaceous vegetation, and shrub in the central east and southeast region (Hanberry, 2020a). Frequent fire was the primary historical disturbance in the eastern and central United States that controlled tree densities, with support from deer, after extinction of more than 20 large megafauna, including species much larger than deer such as proboscideans (i.e., mammoth, mastodon, gomphothere) about 13,000–11,000 years ago following arrival of humans (Means, 2006). Fires maintained grasslands in the central United States, which now are being invaded by eastern tree species such as eastern redcedar and broadleaf species, and likewise, fires maintained oak and/or pine open forests in the eastern United States against forest closure (Hanberry, 2020b; Hanberry et al., 2018). With fire exclusion and at current deer densities, deer have not been able to transition closed forests back to open forests, at least not at landscape levels, nor do tree species increases or decreases match with deer browse preferences (Hanberry & Abrams, 2019).

Due particularly to deer preference for forests, deer browsing on trees directly kills small trees or reduces their growth. About 59% of 74 million forestland ha had moderate or high browse impacts in the northern half of the eastern and central United States (McWilliams et al., 2018). Nonetheless, for the forest products industry, which primarily is based on pine production, deer and other large herbivores help provide control of tree species competitors, which are treated by expensive applications of chemical and mechanical treatments (De Vriendt et al., 2020; Stokely & Betts, 2020; Warren & Hurst, 1981). Additionally, some evidence suggests that large herbivores may increase the growth rate of more mature trees (Lucas et al., 2013; Thompson & Curran, 1993). Secondly, deer help with fuel reduction, which is the primary way to prevent severe fires (Jones et al., 2017). Wildfires do occur in the eastern United States even though the humid climate and limited number of extreme fire weather days greatly reduce fire risk compared to the western United States; moreover, extreme fire weather days are expected to increase with climate change (Hanberry, 2020a, 2020c). Finally, reduction of small diameter trees is the primary requirement for ecological restoration and management of open forests, which support a range of biodiversity that current closed forests are failing to do (e.g., herbaceous plants and early successional bird species; Hanberry et al., 2020; Hanberry & Thompson, 2019; Miller, 1963). Furthermore, an open midstory is important for wildlife maneuverability (i.e., reduced forest “clutter”), in particular, foraging space for some bat species. Open forest management focuses on control of small diameter trees to allow coexistence of a diverse herbaceous ground layer that, in addition to maintaining herbaceous plants, provides resources to associated biodiversity (Bragg et al., 2020).

## 5 CONCLUSIONS

According to relationships between deer densities and land cover, this study substantiated that deciduous broadleaf forest overall was the most influential and positive variable for greater deer densities. Due to their preference for forests, deer damage forest products, but also deer may restore a little balance between herbaceous plants and woody plants by reducing densities of small diameter trees. In contrast to the view that deer are harmful, after extinction of other large mammalian herbivores and exclusion of surface fire that historically reduced tree densities, deer could be considered a favorable disturbance for tree reductions and continued forb presence with associated biodiversity. This modeling approach did not detect benefits of agriculture for deer density at landscape scales, although additional study is warranted. Residential development also appeared to have at least weakly negative relationships with deer density. Agricultural conversion and intensification along with urbanization may help explain recent slight declines in white‐tailed deer populations (Adams & Ross, 2015), making these results useful for large‐scale deer management.

## CONFLICT OF INTEREST

The author has no conflicts of interest.

## AUTHOR CONTRIBUTION

**Brice B. Hanberry:** Conceptualization (lead); Data curation (lead); Formal analysis (lead); Methodology (lead); Project administration (lead).

## Data Availability

The National Land Cover Database is available at https://www.mrlc.gov/. Deer densities are available at https://www.fs.usda.gov/rds/archive/catalog/RDS‐2019‐0053.
